# Supplementary figures and images for: Down-Regulation of miR-194-5p for Predicting Metastasis in Breast Cancer Cells
Source: Int J Mol Sci. 2021 Dec 28;23(1):325. doi: 10.3390/ijms23010325 (PMC8745262; doi:10.3390/ijms23010325)

Figure S1

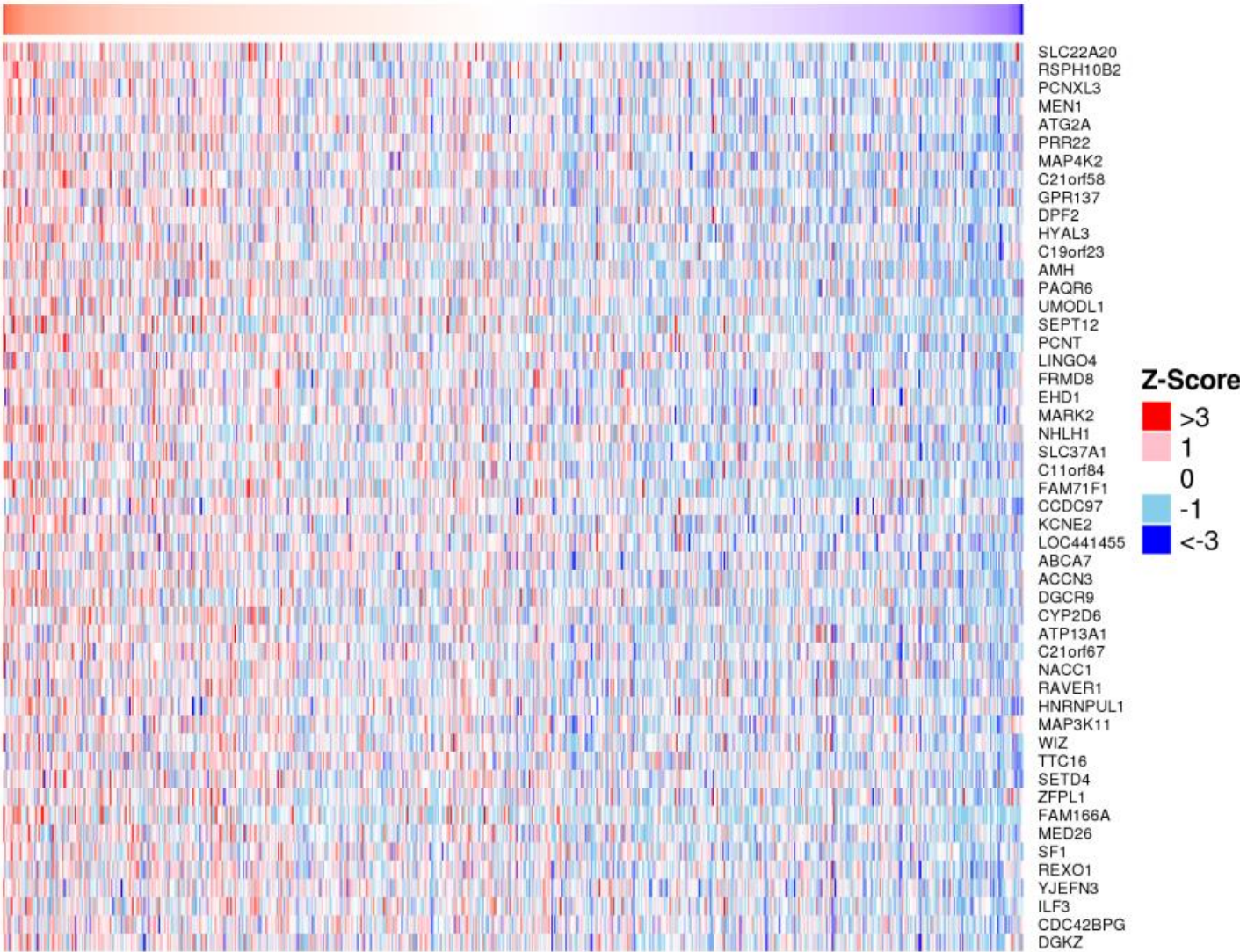

Figure S2

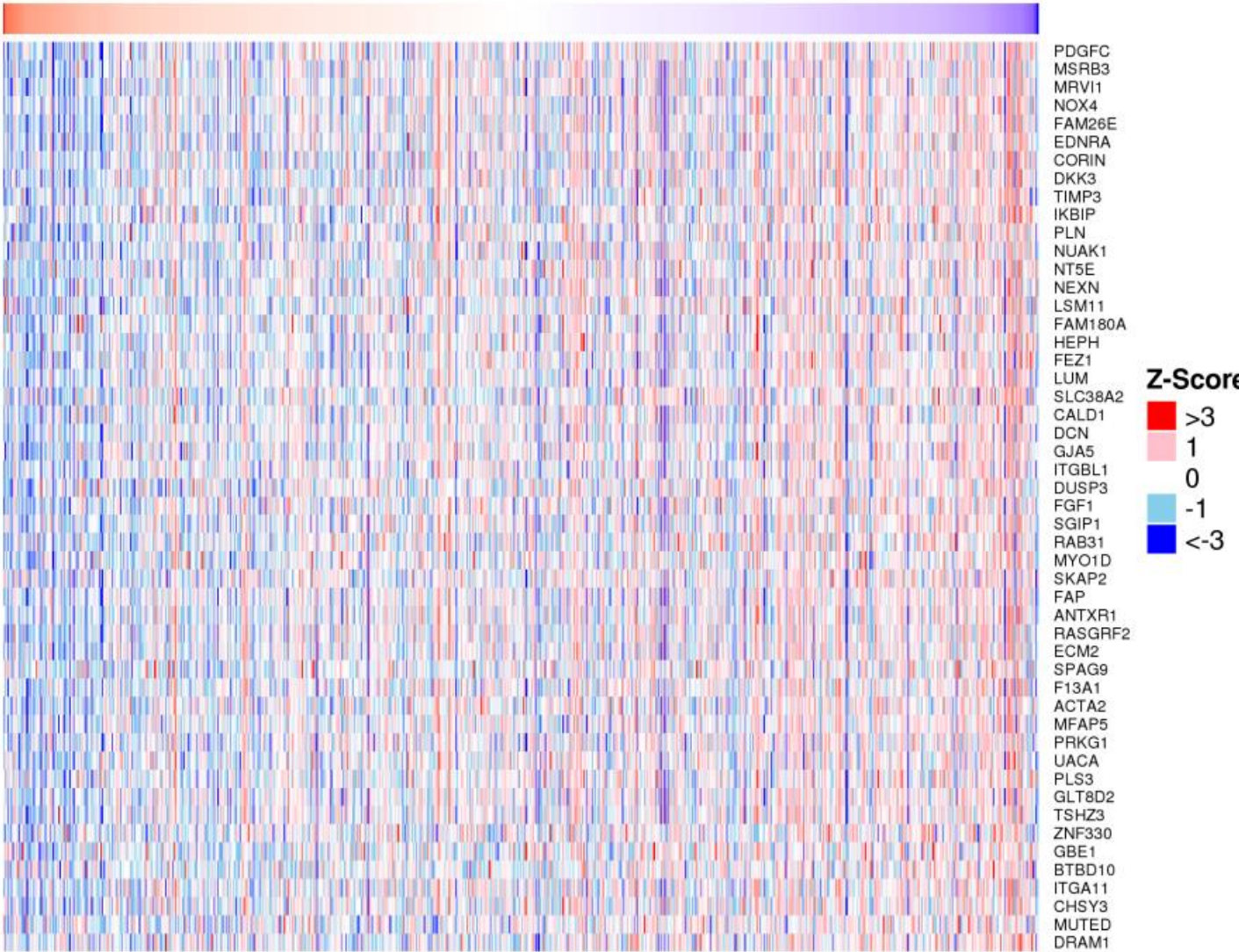

Supplement: Supplementary file 1 [file ijms-23-00325-s001.zip › ijms-1485874-supplementary.pdf]
